# Production of Functional Human Vitamin A Transporter/RBP Receptor (STRA6) for Structure Determination

**DOI:** 10.1371/journal.pone.0122293

**Published:** 2015-03-27

**Authors:** Conor J. Breen, Darren S. Martin, Hui Ma, Kate McQuaid, Richard O’Kennedy, John B. C. Findlay

**Affiliations:** 1 Department of Biology, National University of Ireland Maynooth, Maynooth, Co. Kildare, Ireland; 2 National Centre for Sensor Research, Biomedical Diagnostics Institute, Dublin City University, Dublin, Ireland; Duke University, UNITED STATES

## Abstract

STRA6 is a plasma membrane protein that mediates the transport of vitamin A, or retinol, from plasma retinol binding protein (RBP) into the cell. Mutations in human STRA6 are associated with Matthew-Wood syndrome, which is characterized by severe developmental defects. Despite the obvious importance of this protein to human health, little is known about its structure and mechanism of action. To overcome the difficulties frequently encountered with the production of membrane proteins for structural determination, STRA6 has been expressed in *Pichia pastoris* as a fusion to green fluorescent protein (GFP), a strategy which has been a critical first step in solving the crystal structures of several membrane proteins. STRA6-GFP was correctly targeted to the cell surface where it bound RBP. Here we report the large-scale expression, purification and characterisation of STRA6-GFP. One litre of culture, corresponding to 175 g cells, yielded about 1.5 mg of pure protein. The interaction between purified STRA6 and its ligand RBP was studied by surface plasmon resonance-based binding analysis. The interaction between STRA6 and RBP was not retinol-dependent and the binding data were consistent with a transient interaction of 1 mole RBP/mole STRA6.

## Introduction

The vitamin A transporter/retinol binding protein (RBP) receptor, alternatively called STRA6 (Uniprot: Q9BX79), is an integral plasma membrane protein that mediates the bidirectional transfer of retinol, or vitamin A, between plasma RBP (Uniprot: P02753) and the intracellular retinoid-handling protein machinery [1, 2]. In plasma, RBP circulates in a non-covalent complex with transthyretin (TTR), which stabilizes the binding of retinol to RBP [3, 4]. On binding of RBP to STRA6, retinol is transported into the cell, but the RBP is not internalised [1, 5, 6]. Although seen in bacteria [7, 8], this type of double-function as a receptor and transporter is unique in eukaryotic systems. Mutations in human STRA6 can cause Matthew-Wood syndrome, which is characterized by variable combinations of severe developmental defects such as microphthalmia/anophthalmia, cardiac abnormalities, pulmonary dysplasia and diaphragmatic hernia [9–11]. However, such severe dysfunctional abnormalities are not observed in individuals lacking an active RBP [12, 13] or in a receptor knock-out mouse model [14–16]. This has raised the possibility that there are other, as yet undiscovered, roles that the receptor may play in humans. Initiation of a signalling cascade has been put forward, particularly in the context of insulin resistance and type II diabetes [15, 17, 18], though it remains to be substantiated [16, 19]. Thus signal transduction adds a further dimension to an already novel protein.

The evolutionary development of STRA6 is also opaque. Based on sequence comparisons, there are no other obvious family members in the human genome such as might be seen with other lipocalin receptors, for example the lipocalin 1-interacting membrane receptor (LIMR), NGALR, CD45 and megalin (reviewed in [20]). Nor is there any clear ancestral relationship with any other gene/protein [21] other than another RBP receptor in the liver [22]. This markedly hampers our understanding both of its structure and the mechanisms of its several actions. Clearly, therefore, a considerable degree of understanding could arise, exceptionally, from the structural and functional characterization of the isolated protein. Thus far, the only information available regarding the structure of STRA6 is the topology of the protein, with experimental evidence to date suggesting nine transmembrane segments, an extracellular N-terminus and an intracellular C-terminus [23]. However, no data are available on the secondary, tertiary or quaternary structure of the receptor. Eukaryotic membrane proteins are notorious for presenting significant problems for production in amounts suitable for structural studies and for their stability when removed from their natural lipid environment.

This paper describes the overexpression, isolation, stabilization and characterization of the active receptor as a GFP fusion protein. This strategy greatly facilitates the production of membrane proteins for structural studies. By monitoring the fluorescence of the GFP fusion, the expression, sub-cellular location, detergent-solubilisation efficiencies and monodispersity of the fusion protein can be readily assessed. The fluorescence itself gives a good indication that the receptor is correctly folded. The GFP tag can then be removed using HRV 3C protease for structure determination. Indeed, the vast majority of recently-solved membrane protein structures have used the GFP-fusion approach. [24].

## Results and Discussion

### Generation of a strain of *Pichia pastoris* that over-expresses STRA6-GFP

**Fig 1:**
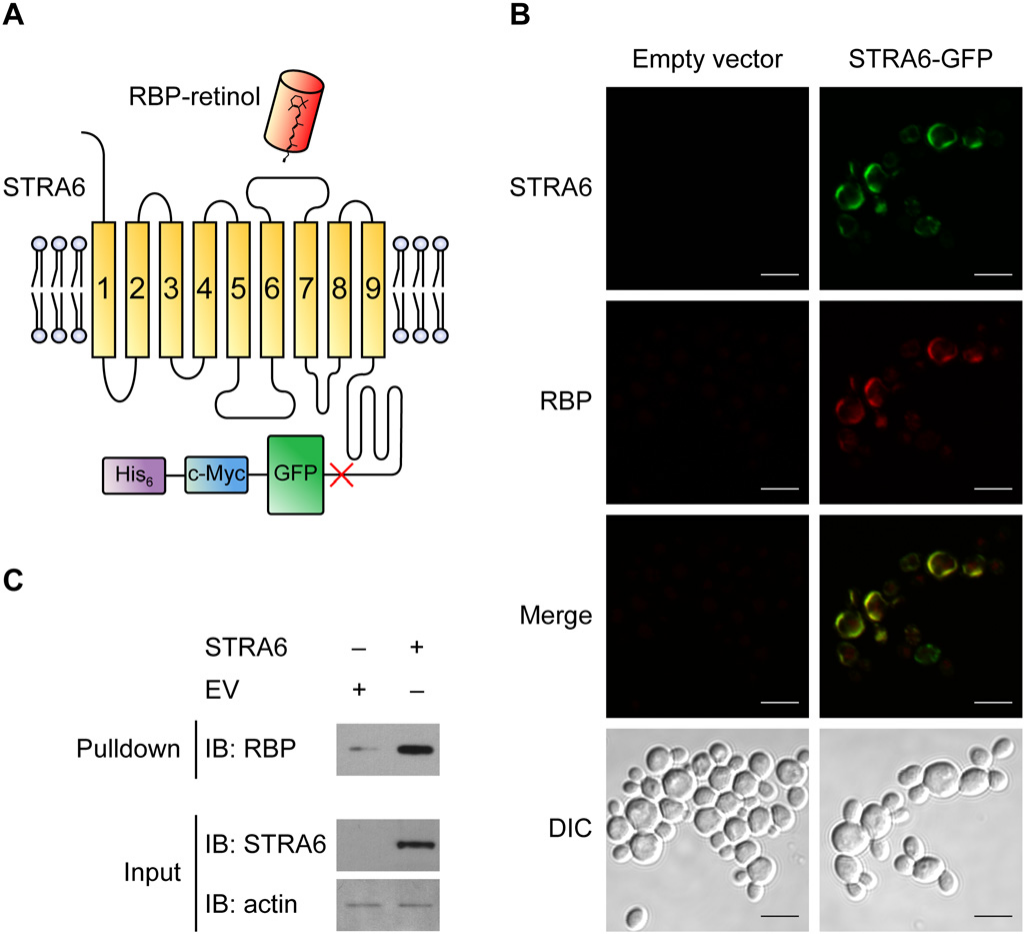
Functional expression of STRA6-GFP in *Pichia pastoris*. **(A)** Schematic of the STRA6-GFP-c-Myc-His_6_ construct designed in this study. The location of a HRV 3C protease cleavage site is indicated by the red cross. **(B)** Co-localization of STRA6-GFP and DyLight594-conjugated holo-RBP at the cell surface of *Pichia pastoris* as determined by confocal microscopy. Top panel: GFP fluorescence, indicating the location of STRA6-GFP at the cell surface. Second panel: DyLight-594 fluorescence, showing the binding of RBP to the surface of cells transformed with STRA6-GFP but not empty vector. Third panel: merged image showing the colocalization of STRA6-GFP and DyLight-594 RBP at the cell surface. Bottom panel: Differential interference contrast (DIC) image of the yeast cells. Scale bar: 5 μm **(C)** Co-purification of holo-RBP with broken cells isolated from yeast transformed with STRA6-GFP but not with broken cells from empty-vector-transformed cells.

A STRA6 construct with a cleavable GFP fusion protein was designed for this study (Fig 1A) and cloned into the pPICZ-A expression vector. A clone of *P*. *pastoris* expressing high levels of STRA6-GFP (a “jackpot” clone) was obtained using a post-transformational vector amplification (PTVA) approach [25]. In this method, transformants were re-streaked on plates containing progressively higher levels of antibiotic, *i*.*e*. 100 μg/mL, 500 μg/mL, 1 mg/mL and 2 mg/mL Zeocin. This technique is reported to select for transformants containing multiple copies of the gene of interest [25]. The clone selected demonstrated strongly-enhanced fluorescence due to STRA6-GFP when compared to other clones, as determined by fluorescence microscopy.

### STRA6-GFP is expressed at the cell surface and binds RBP

STRA6-GFP was predominantly expressed at the cell surface (Fig 1B) indicating that the fusion protein had reached its correct subcellular destination. Additionally, the observation that STRA6-GFP was fluorescent is a reliable indicator that the fusion protein had integrated correctly into the membrane [26, 27]. Encouragingly, excellent correlation has been found between the activity of transporter proteins and the fluorescence of their GFP fusion constructs [28].

To test whether STRA6-GFP could bind its ligand, RBP, co-localization analysis was performed using confocal microscopy. RBP was expressed in *Pichia pastoris*, purified as described previously [29] and labelled with DyLight 594 for the microscopy studies (see also S1 Fig). DyLight 594-conjugated holo-RBP was found associated only with the surface of cells that were transformed with STRA6-GFP indicating that the recombinant fusion protein is functional *in situ* (Fig 1B). Furthermore, holo-RBP co-purified with broken cells prepared from cells transformed with STRA6-GFP but not with broken cells prepared from cells transformed with empty vector (Fig 1C).

### STRA6-GFP is monodisperse in a range of detergents used for crystallization

The analysis of the monodispersity and stability of the membrane protein under investigation in different detergents is a critical step on the road to structural determination. The GFP-fused membrane protein was solubilized using a range of detergents, Ni-NTA purified and subjected to fluorescence-detection size exclusion chromatography (FSEC) [30]. Detergent concentrations for solubilisation and size-exclusion chromatography were based on the recommendations of Hays *et al*., 2010 [31].

**Fig 2:**
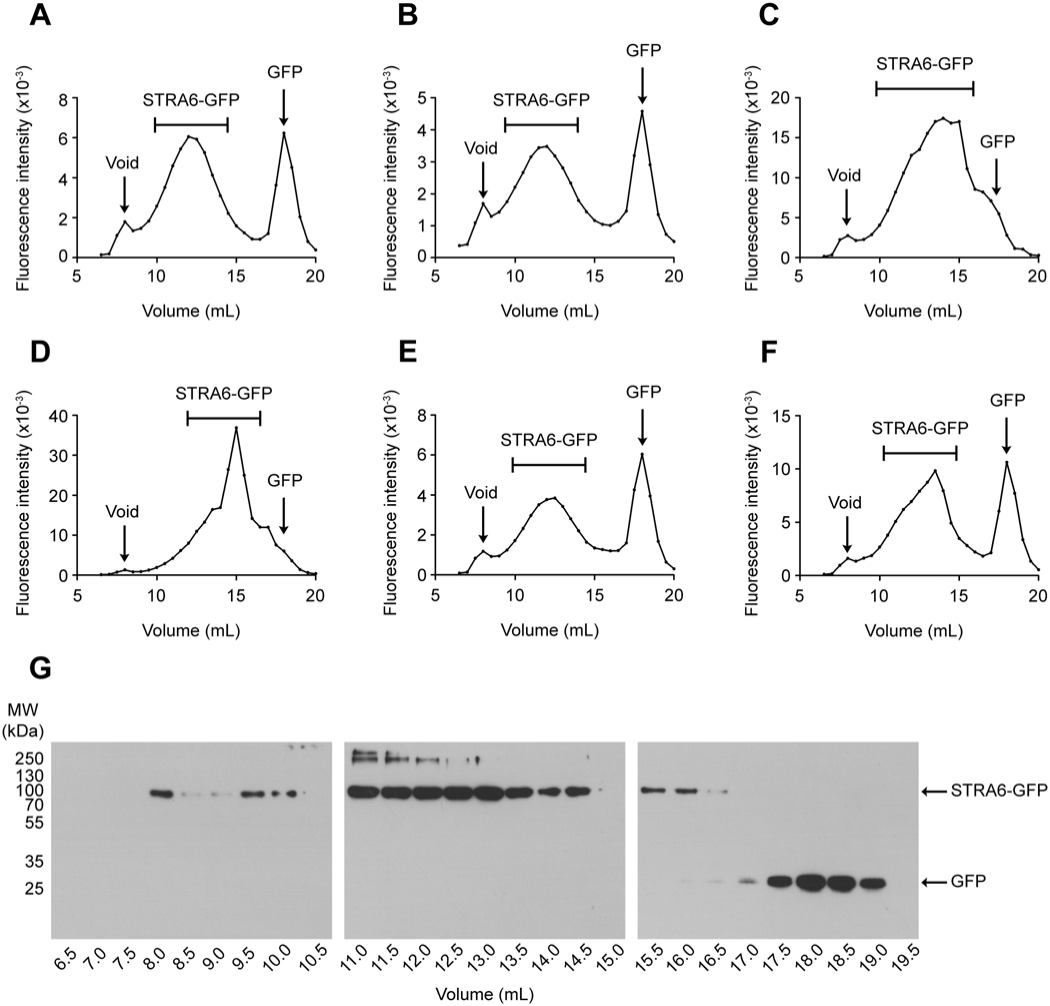
Detergent screening of STRA6-GFP by FSEC. The dispersity of STRA6-GFP was determined on a Superose 6, 10/300 GL column by FSEC in the following detergent complexes: **(A)** C12E9, **(B)** DM, **(C)** LDAO, **(D)** FC-12, **(E)** DDM, **(F)** DDM:CHS. The peaks corresponding to the void volume, the STRA6-GFP fusion protein and free GFP are indicated in the elution profiles. The void volume was determined using the elution profile of blue dextran. **(G)** The positions of the STRA6-GFP fusion protein (~105 kDa) and free GFP (~30 kDa) were determined by immunoblotting across the elution profile of STRA6-GFP in DDM using an anti-c-Myc antibody.

Encouragingly, STRA6-GFP eluted as a single broad symmetrical peak in C12E9 (0.05%), DM (0.2%) and DDM (0.05%) from an analytical size-exclusion column (Fig 2A, 2B and 2E). All of these detergents are non-ionic and have been successfully used to crystallize membrane proteins [32, 33]. The broad peaks observed for STRA6-GFP in these detergents, when compared to that observed for GFP, may indicate the presence of multiple oligomeric forms of the receptor. Alternatively, the presence of lipid molecules could contribute to the broad peaks observed for the fusion protein. Another contributing factor may be that STRA6 itself may not have a uniform Stokes radius due to an elongated structure, particularly if the extensive C-terminal domain is attached to the membrane sector by a flexible region.

STRA6-GFP was not monodisperse in the zwitterionic detergents LDAO (0.28%; Fig 2C) and FC-12 (0.14%; Fig 2D) although these latter harsher detergents solubilized STRA6-GFP more effectively than their non-ionic counterparts (S2 Fig). Interestingly, a sharp peak of STRA6-GFP is observed in the presence of FC-12, which may indicate the disruption of an oligomer. It is perhaps unsurprising that STRA6-GFP was not monodisperse in LDAO, since it has been estimated that only around 20% of membrane proteins are stable in this detergent despite being one of the most successful detergents for membrane protein crystallization [34, 35]. However, a study has shown that medium to high resolution crystal structures were found to be more likely in bacterial transporters that were resistant to unfolding and subsequent aggregation in LDAO [36]. It is interesting to note that none of the conditions tested lead to a significant proportion of aggregated STRA6-GFP in the void volume, which may indicate that this is a very stable receptor and suitable for future structural studies.

The cholesterol analogue cholesteryl hemisuccinate (CHS), particularly in a complex with DDM, has been successfully used to stabilize integral membrane proteins, such as GPCRs [37, 38]. Considering STRA6-GFP was monodisperse in DDM alone, it is surprising that STRA6-GFP was not monodisperse in DDM/CHS (0.05%:0.01%; Fig 2F) although a higher yield of protein was achieved with DDM/CHS than was achieved with DDM alone (S2 Fig). It is possible therefore that CHS stabilizes a particular oligomeric form of STRA6-GFP. Since it is unclear how many molecules of CHS are binding to the receptor in solution, it would be interesting to test whether increasing either the ratio of CHS to DDM, or increasing the total amount of DDM/CHS used to solubilize the receptor could further stabilize this oligomeric form.

### Large scale purification of STRA6-GFP from fermentor-grown cells

**Fig 3:**
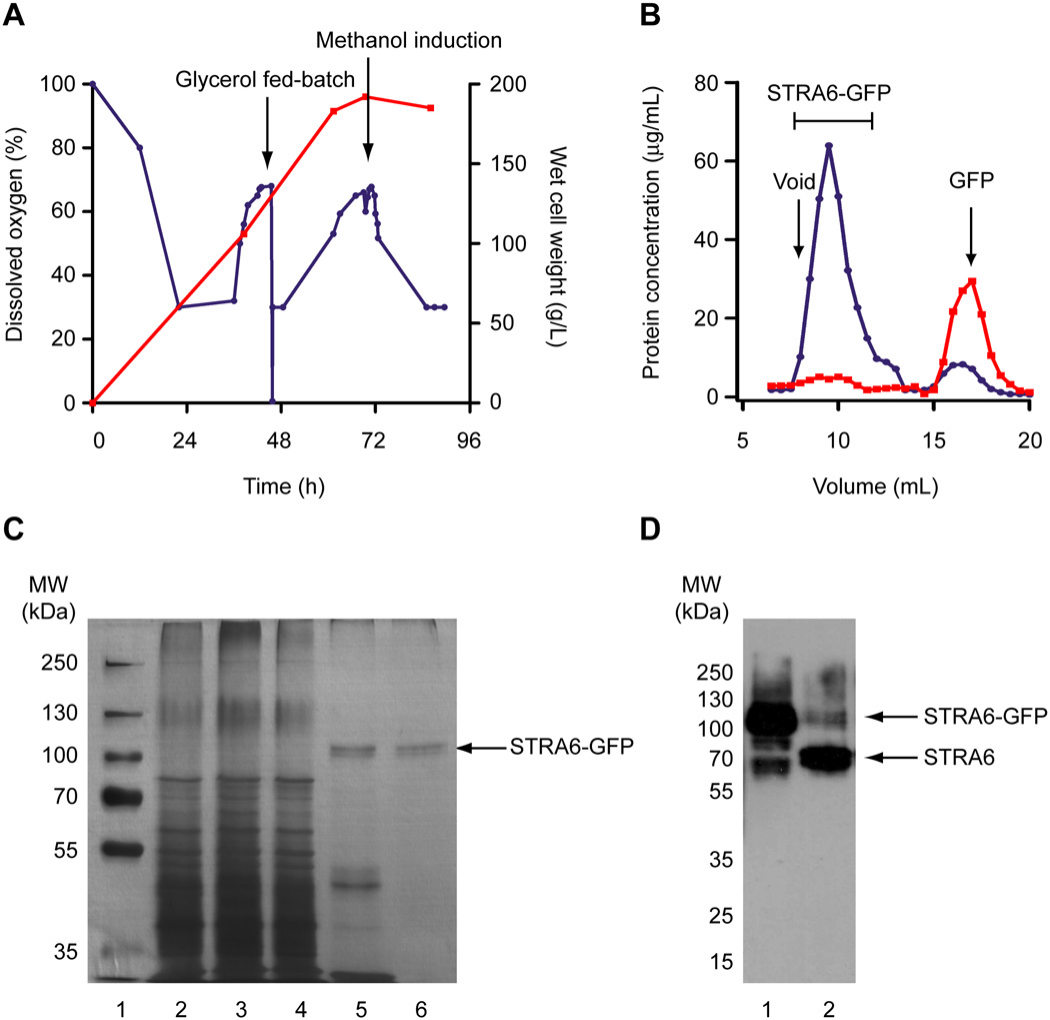
Purification of STRA6 from *Pichia pastoris*. **(A)** Fed-batch fermentation of recombinant *Pichia pastoris* expressing STRA6-GFP. Changes in the wet cell weight of yeast (red line) and dissolved oxygen (blue line) are shown over time. **(B)** Elution profile of Ni-NTA-purified STRA6-GFP on a Superdex 200, 10/300 GL column (blue trace) and after removal of GFP by treatment with HRV 3C protease (red trace). The disappearance of the peak corresponding to STRA6-GFP and appearance of a peak corresponding to GFP are clearly visible. The concentration of protein was determined by comparing the fluorescence of the eluted fractions to a standard curve of GFP and correcting for the molecular weight of the fusion protein. The void volume was determined using the elution profiles of blue dextran. **(C)** A silver-stained 10% SDS gel showing the purification of STRA6-GFP. The lanes are: *lane 1*, MW markers; *lane 2*, microsomes, 10 μg; *lane 3*, solubilized microsomes after extraction with C12E9, 10 μg; *lane 4*, flow-through from Ni-NTA column, 10 μg; *lane 5*, 250 mM imidazole eluate from Ni-NTA column, 3 μg; *lane 6*, eluate from Superdex 200, 10/300 GL column, 3 μg. **(D)** Immunoblot of STRA6-GFP after purification by Ni-NTA and size exclusion chromatography (lane 1) and following treatment with HRV 3C protease to remove the GFP (lane 2) using an antibody against full-length STRA6. The positions corresponding to the predicted molecular weights of STRA6-GFP and STRA6 are indicated.

In order to generate enough STRA6 to perform kinetic and equilibrium-binding studies, a PTVA-selected clone of STRA6-GFP was grown in the fermentor. Cells were harvested 48 hours after methanol induction. Approximately 1 kg of yeast was harvested from a 5.5 L fermentor culture (Fig 3A). Cells were washed once in PBS, transferred to 50 mL falcons and re-centrifuged. Yeast pellets were then extruded into liquid nitrogen through a hole punched in the bottom of the tube using a plunger from a 60 mL disposable syringe. The resulting “spaghettis” were cryogenically pulverized using the Retsch Mixer Mill 400 as described in “Methods”.

Following pulverization, microsomes were prepared using the method of Lerner-Marmarosh *et al*. (1999) with slight modifications [39]. It was found, for example, that supplementation of the resuspension buffer with 1 mM EDTA dramatically reduced proteolysis of STRA6-GFP. Although EDTA is incompatible with downstream Ni-NTA purification, we reasoned that it would be removed by centrifugation during the preparation of microsomes. By comparing the fluorescence of solubilized microsomes to a standard curve of GFP,. it was estimated that these microsomes contained 5 mg of GFP equivalent per 100 g of original wet cell weight of yeast.

The strategy used for detergent solubilisation of STRA6-GFP from microsomes was based on two observations about the behaviour of the receptor in the detergents analysed. Firstly, STRA6-GFP ran as a symmetrical, monodisperse peak in three out of the six detergent mixtures tested, namely C12E9, DDM and DM (Fig 2). Of these detergents, C12E9 provided a higher yield of the receptor based on a comparison of the area under the curves from the FSEC elution profiles (Fig 2). Secondly, STRA6-GFP displayed very different behaviour when analysed by SDS-PAGE depending on the detergent in the protein-detergent complex. Of the three aforementioned detergents, only STRA6-GFP in DDM showed an appreciable level of monomer when analysed by SDS-PAGE, making it difficult to estimate the purity of the receptor when complexed with C12E9 or DM. However, when STRA6-GFP was solubilized in C12E9 and the detergent exchanged with DDM during purification on Ni-NTA, a large proportion of STRA6-GFP was found to run as a monomer when analysed by SDS-PAGE. Therefore, by solubilizing STRA6-GFP in C12E9 and exchanging the detergent with DDM on Ni-NTA, the receptor ran as a symmetrical, monodisperse peak on a Superdex 200, 10/300 GL column and migrated at ~100 kDa by SDS-PAGE (Fig 3B and 3C). This band was excised from the gel, digested with trypsin and analysed by mass spectrometry, which confirmed that the protein was STRA6-GFP (Tables 1 and 2). Using this purification strategy, we reproducibly obtained about 1.0 mg of highly-purified fusion protein from 120 g of yeast (wet cell weight).

**Table 1:** Mass spectrometry of STRA6-GFP.

Peptide sequence	Mr expt (Da)	Mr calc (Da)	Location, Position	MS/MS
GRPGLPSPVDFLAGDRPR	1907.0482	1906.0173	IC1	+
GLQSSYSEEYLR	1430.7854	1430.6677	IC3	+
HGFLSWAR	972.5854	972.4930	IC3	+
AGVTTDVSYLLAGFGIVLSEDK	2254.2254	2254.1733	EC3	+
GAALDLSPLHR	1148.5654	1148.6302	IC4	+
VLLSALYNAIHLGQMDLSLLPPR	2550.5482	2549.4039	C-terminus	+
AATLDPGYYTYR	1389.7454	1389.6565	C-terminus	+
TMAAPQDSLRPGEEDEGMQLLQTK	2645.2282	2644.2472	C-terminus	+
WGLAYTLLHNPTLQVFR	2028.3254	2028.0945	C-terminus	+
TALLGANGAQPLEVLFQGPLEMVSK	2584.1482	2583.3850	STRA6, C-term; HRV 3C	-
GEELFTGVVPILVELDGDVNGHK	2437.4454	2436.2537	GFP	+
FSVSGEGEGDATYGK	1502.8054	1502.6525	GFP	+
SAMPEGYVQER	1265.7254	1265.5710	GFP	+
TIFFKDDGNYK	1347.2782	1346.6507	GFP	+
FEGDTLVNR	1049.6054	1049.5142	GFP	+
GIDFKEDGNILGHK	1542.3982	1541.7838	GFP	+
LEYNYNSHNVYIMADK	1989.0682	1988.8938	GFP	+
DHMVLLEFVTAAGITLGMDELYK	2566.1782	2565.2859	GFP	+
LISEEDLNSAVDHHHHHH	2126.2882	2126.9750	c-Myc; hexahistidine	-

The sequence coverage of STRA6-GFP based on tryptic peptides identified by LC-MS/MS is shown.

**Table 2:** Sequence of STRA6-GFP showing identified fragments underlined in bold text.

MSSQPAGNQTSPGATEDYSYGSWYIDEPQGGEELQPEGEVPSCHTSIPPGLYHACLASLSILVLLLLAMLVRRRQLWPDCVR**GRPGLPSPVDFLAGDRPR**AVPAAVFMVLLSSLCLLLPDEDALPFLTLASAPSQDGKTEAPRGAWKILGLFYYAALYYPLAACATAGHTAAHLLGSTLSWAHLGVQVWQRAECPQVPKIYKYYSLLASLPLLLGLGFLSLWYPVQLVRSFSRRTGAGSK**GLQSSYSEEYLR**NLLCRKKLGSSYHTSK**HGFLSWAR**VCLRHCIYTPQPGFHLPLKLVLSATLTGTAIYQVALLLLVGVVPTIQKVR**AGVTTDVSYLLAGFGIVLSEDK**QEVVELVKHHLWALEVCYISALVLSCLLTFLVLMRSLVTHRTNLRALHR**GAALDLSPLHR**SPHPSRQAIFCWMSFSAYQTAFICLGLLVQQIIFFLGTTALAFLVLMPVLHGRNLLLFRSLESSWPFWLTLALAVILQNMAAHWVFLETHDGHPQLTNRRVLYAATFLLFPLNVLVGAMVATWR**VLLSALYNAIHLGQMDLSLLPPRAATLDPGYYTYR**NFLKIEVSQSHPAMTAFCSLLLQAQSLLPR**TMAAPQDSLRPGEEDEGMQLLQTK**DSMAKGARPGASRGRAR**WGLAYTLLHNPTLQVFRKTALLGANGAQPLEVLFQGPLEMVSKGEELFTGVVPILVELDGDVNGHKFSVSGEGEGDATYGK**LTLKFICTTGKLPVPWPTLVTTLTYGVQCFSRYPDHMKQHDFFK**SAMPEGYVQERTIFFKDDGNYK**TRAEVK**FEGDTLVNR**IELK**GIDFKEDGNILGHKLEYNYNSHNVYIMADK**QKNGIKVNFKIRHNIEDGSVQLADHYQQNTPIGDGPVLLPDNHYLSTQSALSKDPNEKR**DHMVLLEFVTAAGITLGMDELYK**GGRQLGPEQK**LISEEDLNSAVDHHHHHH**

To demonstrate the suitability of the receptor for structural studies, STRA6 has been further isolated by HRV 3C-mediated cleavage of the fusion protein and subsequent removal of GFP by size-exclusion chromatography. FSEC clearly demonstrates disappearance of the fluorescent peak corresponding to STRA6-GFP following treatment with HRV 3C protease and a concomitant increase in the fluorescent peak corresponding to GFP (Fig 3B). Immunoblotting with an anti-STRA6 antibody clearly demonstrates the disappearance of a band at ~105 kDa after treatment with HRV 3C protease and appearance of a band at ~72 kDa corresponding to the removal of the 30 kDa GFP fragment containing the myc and His_6_ epitope tags (Fig 3D).

### SPR experiments reveal transient binding of RBP by STRA6-GFP

**Fig 4:**
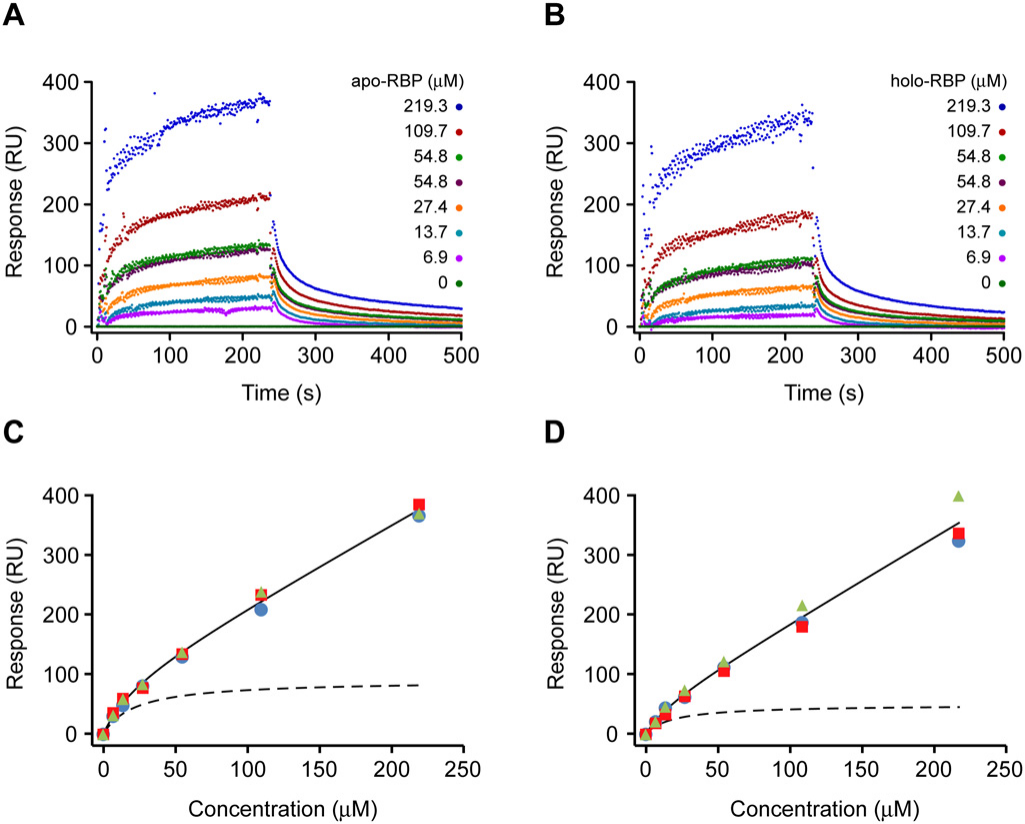
Interaction of apo-/holo-RBP with STRA6 determined by surface plasmon resonance (SPR). Representative SPR data for the binding of various concentrations of **(A)** apo-RBP and **(B)** holo-RBP to immobilized STRA6-GFP. Re-plot of triplicate independent steady-state responses at various concentrations of **(C)** apo-RBP and **(D)** holo-RBP against the total concentration of RBP added. The solid lines represent the total binding of RBP to the receptor as estimated from the solution to equation (1). The dashed lines represent the specific binding of RBP to the receptor without the contribution from non-specific binding. This component was derived by plotting only the specific component of the non-linear regression solution, *i*.*e*. ($\frac{R_{\max}\cdot [L]}{K_{D}+[L]}$) from equation (1).

Surface plasmon resonance (SPR) experiments were used to study the binding of apo- and holo-RBP to a highly-purified preparation of detergent-solubilized STRA6-GFP. The purified receptor was immobilized via its C-terminal c-Myc tag to a flow-cell containing an immobilized anti-c-Myc antibody. A concentration-dependent increase in response units was observed when either apo-RBP or holo-RBP was injected onto the flow-cell containing STRA6-GFP and corrected for non-specific binding using a flow-cell containing an immobilized anti-HA antibody as a control (Fig 4A and 4B). The association and dissociation components were both biphasic, confirming the previous observations of Sivaprasadarao and Findlay [40].

To estimate the K_D_ from all of the triplicate independent steady-state responses for the binding of both apo- and holo-RBP to STRA6-GFP (Fig 4C and 4D), a one-site binding model with a linear component to correct for low-affinity binding was employed:

$$bound=\frac{R_{\max}\cdot [L]}{K_{D}+[L]}+m\cdot [L]$$ (1)

where *R*_*max*_ is the maximum specific binding of the ligand, *K*_*D*_ is the equilibrium binding constant, *[L]* is the ligand (analyte) concentration and *m* is the slope of the linear component for low-affinity binding. Interestingly, the affinities of apo- and holo-RBP for STRA6-GFP were both in the micromolar range: K_D_ = 22.4±8.1 µM (mean±S.E., P-value = 0.013) for apo-RBP binding and K_D_ = 19.4±3.9 µM (mean±S.E., P-value = 0.008) for holo-RBP binding. No statistical difference was observed between the binding of apo- and holo-RBP to the receptor using a two-tailed, independent t-test to compare both pairs of triplicate equilibrium constants derived from the independent response curves (P = 0.31).

The equilibrium constants obtained in this study contrast with two earlier studies which have indicated that RBP and the RBP receptor interact with nanomolar affinity. In the first of these studies, the authors used a centrifugation-based assay to measure the rates of association and dissociation of RBP with membranes prepared from placental microvilli and demonstrated that both the association and dissociation curves are biphasic, with very fast- and slow-binding and release components [40]. However, due to the inherent limitations of the assay, specifically, the time taken to separate free RBP from membranes by centrifugation, the fast components of association and dissociation, which the authors acknowledged were low-affinity binding sites, could not be measured. Instead, these components were reduced from an exponential-containing term to a constant so that the slow rate constants could be estimated from the slow components of association and dissociation. In this study, we confirm that both the association and dissociation of RBP and its receptor are biphasic but have chosen instead to re-plot the steady-state RBP binding responses against the total concentration of RBP added, which avoids the complexity of estimating equilibrium binding constants from multiple rate constants. It should also be remembered that the original studies were carried out with native membranes, conditions which could have a significant effect on the behaviour of the receptor.

In another study that estimated the affinity of RBP for its receptor, the authors measured the binding of different concentrations of an alkaline phosphatase (AP)-RBP fusion protein to STRA6-transfected cells using a colorimetric assay [1]. In this assay, the initial reaction of alkaline phosphatase with its substrate, BCIP, is irreversible, forming a dark blue precipitate after hydrolysis. Since the reaction of alkaline phosphatase with BCIP can only proceed in the forward direction, this reaction is unsuitable for making assumptions about equilibrium dynamics, a process which operates in both the forward and reverse direction.

### Receptor Occupancy by RBP

The equilibrium binding constants for the binding of apo- and holo-RBP to STRA6-GFP can be used to estimate the fractional receptor occupancy (ϑ) of STRA6 under physiological conditions, according to the equation:

$$\theta =\frac{\left[L\right]}{[L]+K_{D}}$$ 2

Based on a plasma RBP concentration of ~1.1 μM [41], the fractional receptor occupancy of STRA6 would be approximately 5%. However, it is likely that the true fractional occupancy is modulated by the association of RBP with TTR under physiological conditions. Previous experiments demonstrated that RBP binding to its receptor was inhibited by the presence of TTR [40], subsequently explained by the observation that the interaction site between RBP and TTR produced steric inhibition of the interaction between RBP and the receptor [42, 43]. Therefore the effective native fractional occupancy based on the concentration of free RBP in plasma would be much lower.

SPR can also be used to determine the stoichiometry of protein-protein interactions, since the signal is proportional to the mass of protein in the vicinity of the sensor surface [44, 45]. Thus, the stoichiometry of the binding of RBP to STRA6-GFP can be estimated from the following equation:

$$stoichiometry=\frac{B_{\max}\cdot MW_{R}}{MW_{L}\cdot B_{R}}$$ 3

where *B*_*max*_ is the maximum specific binding of the ligand, *MW*_*R*_ is the molecular weight of the receptor, *MW*_*L*_ is the molecular weight of the ligand and *B*_*R*_ is the binding of receptor to the chip. Using this formula, a stoichiometry of 0.71 ± 0.22 (mean ± s.d.; n = 3) was found for apo-RBP binding to STRA6-GFP while a stoichiometry of 0.64 ± 0.25 (mean n ± s.d.; n = 3) for the binding of holo-RBP to STRA6-GFP. Given these data, a stoichiometry of 1:1 seems likely. These finding are consistent with a single binding site for RBP on the third extracellular loop of STRA6 [46]. Clearly, further work will need to be carried out to determine whether STRA6 exists as a monomer or multimeric complex and to resolve the architecture of the channel through the plasma membrane. If STRA6 does indeed exist as a multimer, it would appear that there is little steric competition for the interaction site. However, *in vivo*, due to the low concentration of free RBP, only one molecule/multimer would be bound. A similar situation is seen with the RBP:TTR complex in plasma.

## Conclusions

### Large scale purification of STRA6-GFP

We describe the expression and purification of the RBP receptor and vitamin A transporter, STRA6. A STRA6-GFP fusion protein was expressed in *Pichia pastoris* and was correctly targeted to the cell surface where it was able to bind its ligand, RBP. Pulldown experiments revealed increased co-purification of RBP with broken cell preparations from STRA6-GFP-transformed cells compared to preparations from empty vector-transformed cells, thus confirming functional expression of the receptor.

Expression of STRA6 as a GFP fusion protein enabled the use of FSEC to identify detergents that could effectively solubilize the receptor and maintain it in a stable, monodisperse state, conditions which are key to the successful crystallization of membrane proteins. Using this technique, STRA6 is soluble and monodisperse in DDM, DM and C12E9, all of which have been successfully used to crystallize membrane proteins. Using surface plasmon resonance, the affinity constants of apo- and holo-RBP for STRA6 are in the micromolar range (K_D_ = 22.4 ± 8.1 μM and K_D_ = 19.4 ± 3.9 μM for apo- and holo-RBP binding, respectively) consistent with transient interactions.

A 5.5 L fermentor culture yielded approximately 1 kg of yeast, from which ~8.5 mg of pure, monodisperse STRA6-GFP could be obtained: greater yields could be obtained by further optimizing the expression and purification conditions. Nevertheless, the current yield already permit electron microscopy, crystallography and SEC-MALS (size exclusion chromatography coupled to a multi-angled light scattering detector) to be carried out, from which a wealth of structural and functional information can be obtained.

## Materials and Methods

### Materials

*Pichia pastoris* strain KM71H (aox::ARG4, arg4), expression vector pPICZ-A and Zero Blunt TOPO PCR cloning kit were purchased from Invitrogen. A plasmid containing the gene for full-length human STRA6 transcript variant 2 (NM_022369.3) in the pCMV6-XL4 cloning vector, was purchased from OriGene. The pEGFP-N1 plasmid containing eGFP was purchased from Clontech. Zeocin was from Invivogen. DyLight 594 amine-reactive dye was purchased from Pierce. N-dodecyl β-D maltoside (DDM), N-decyl β-D maltoside (DM) and Fos-Choline-12 (FC-12) were from Anatrace. Cholesteryl hemisuccinate, N, N-dimethyldodecylamine N-oxide (LDAO) and dodecyl nonaoxyethylene ether (C12E9) were from Sigma. HRV 3C protease was from Sino Biological. Polyclonal antibody raised against full-length human STRA6 (#H00064220-D01P) was from Abnova. The Biacore 3000 and instrument-specific consumables (CM5 sensor chip, ethyl(dimethylaminopropyl)-carbodiimide, N-hydroxysulphosuccinimide and ethanolamine) were obtained from GE Healthcare.

### Construction of STRA6-GFP expression vector

E-GFP was amplified using the following oligonucleotides: forward primer 5’- CCGCTCGAGATGGTGAGCAAGGGCGAGGAG-3’ and reverse primer 5’- AATAGTATGCGGCCGCCCTTGTACAGCTCGTCCATGCC-3’. The forward primer included the sequence for the XhoI restriction site (underlined) while the reverse primer contained the restriction site for the NotI restriction enzyme (underlined). The PCR product was inserted into the pPICZ-A vector upstream of the c-Myc and His_6_ epitope tags. The sequence for full-length STRA6 was amplified using the following oligonucleotides: forward primer 5’- GAATTCAATTAGTATGTCGTCCCAGCCAGC-3’ and reverse primer 5’- GGACTCGAGGGGCCCCTGGAACAGAACTTCCAGGGGCTGGGCACCATTGGCACC. The forward primer contained an EcoRI restriction site (underlined) and a ribosome binding site (RBS) while the reverse primer contained the sequence for a HRV 3C protease cleavage site and an XhoI restriction site. The STRA6-HRV 3C PCR product was inserted into the newly-created “eGFP-pPICZ-A” vector. The correct STRA6-HRV3C-eGFP-Myc-His_6_ sequence was confirmed by digestion and automated sequencing.

### Expression of STRA6-GFP in *P*. *pastoris*

Transformation of the cells was carried out according to the manufacturer’s instructions using a MicroPulser Electroporator (BioRad). Transformants expressing high levels of STRA6-GFP were selected by post-transformational vector amplification (PTVA) [25]. Single colonies from YPD plates (1% yeast extract, 2% peptone, 2% dextrose, 2% agar) containing 100 μg/mL Zeocin were picked at random and re-streaked on fresh YPD plates containing 500 μg/mL, 1 mg/mL and finally 2 mg/mL Zeocin. Clones were then screened for expression of STRA6-GFP by fluorescence microscopy. Single colonies from each clone were used to inoculate 10 mL BMGY (1% yeast extract, 2% peptone, 100 mM potassium phosphate, pH 6.0, 1.34% YNB, 4 × 10^-5^% biotin, 1% glycerol) in a 50 mL Erlenmeyer flask. After 48 h shaking at 28°C, the cells were harvested by centrifugation at 2,500 × *g* for 5 min at room temperature, resuspended in 10 mL BMMY (1% yeast extract, 2% peptone, 100 mM potassium phosphate, pH 6.0, 1.34% YNB, 4 × 10^−5^% biotin, 0.5% methanol) and incubated at 28°C with shaking at 200 rpm. Methanol was added at 24 h post-induction to a final concentration of 1.0%. The cells were harvested after 48 h by centrifugation at 2,500 × *g* for 5 min at 4°C. To confirm expression of STRA6-GFP, cells were resuspended in ice-cold PBS and analysed by fluorescence microscopy.

For large-scale expression, fermentation of *Pichia pastoris* was carried out in a 14 L autoclavable vessel attached to a New Brunswick BioFlo 115 control station. A 10 mL BMGY culture was grown overnight at 28°C and subsequently added to 240 mL BMGY, pH 6.0 in a 2 L Erlenmeyer flask. The culture was again incubated overnight at 28°C. This culture was then added to 5 L fermentation media (0.5 M H_3_PO_4_, 6.8 mM CaSO_4_, 104.5 mM K_2_SO_4_, 60.5 mM MgSO_4_.7H_2_O, 73.6 mM KOH, pH 5.0 using NH_4_OH) containing 4% glycerol and 4.35 mL per L PTM_1_ (PTM_1–_24 mM CuSO_4_.5H_2_O, 534 μM NaI, 17.7 mM MnSO_4_.H_2_O, 827 μM Na_2_MoO_4_, 323 μM H_3_Bo_3_, 3.9 mM CoCl_2_, 146.8 mM ZnCl_2_, 234.3 mM FeSO_4_.7H_2_O, 820 μM biotin, 93.3 mM H_2_SO_4_). The dissolved oxygen (DO) concentration was maintained above 30% by aeration and agitation. This ‘glycerol batch phase’ continued until all the glycerol was consumed as indicated by the rise in the concentration of dissolved oxygen (DO ~ 65%). The second ‘glycerol-fed batch phase’ was initiated by addition of 50% w/v glycerol containing PTM_1_ (12 mL/L) at a rate of 18.15 mL/L/h. The glycerol feed was maintained until the DO reached a plateau for 2 h (57%) at which time the ‘methanol-fed batch phase’ was initiated by addition of 100% methanol containing PTM_1_ (12 mL/L) at a rate of 1 mL/L/h. After 1.5 h the rate of methanol feed was increased to 1.1 mL/L/h followed by another increase to 1.2 mL/L/h after 30 min. After another 2 h the rate of methanol feed was increased to 1.3 mL/L/h and left overnight. The next day the rate of methanol feed was increased to 2 mL/L/h for 5 h then increased to 2.4 mL/L/h for 4 h before the final rate of 3 mL/L/h was set. The culture was then left for a further 18 h before cells were harvested.

### Preparation of microsomes

STRA6-GFP-expressing cells were harvested by centrifugation at 2,500 × *g* for 10 min in 50-mL tubes. The supernatants were decanted and a small hole (1.2 mm Ø) was punched in the bottom of the tube using an 18-gauge needle. A plunger from a 60-mL syringe was used to extrude the yeast pellet from the 50-mL tube into liquid nitrogen. The resulting “spaghetti” was stored at −80°C. For cell breakage, frozen “spaghetti” was transferred to 2 × 50 mL stainless steel grinding jars, each containing a pre-cooled stainless steel grinding ball (25 mm Ø), that had been pre-cooled in liquid nitrogen. Grinding was performed in 3–5 cycles of 3 min at 30 Hz using a Retsch Mixer Mill 400. Between each cycle, the jar was cooled in liquid nitrogen. Pulverized yeast was either used immediately or stored at −80°C.

Pulverized yeast was resuspended in extraction buffer (50 mM HEPES, 300 mM NaCl, 10% glycerol, 10 mM β-ME, 2 mM TCEP, 1 mM PMSF, 1 mM EDTA, 1 × SIGMA*FAST* protease inhibitor cocktail; pH 7.5 at 4°C) at 1 g pulverized yeast per 3 mL of extraction buffer. After 60 min continuous stirring at 4°C, the crude lysate was centrifuged at 10,000 × *g* for 20 min at 4°C. The pellets were discarded and the supernatant centrifuged at 150,000 × *g* for 90 min at 4°C. The supernatants were discarded and the pellets containing the microsomes were resuspended in membrane wash buffer (50 mM HEPES, 500 mM NaCl, 10% glycerol, 10 mM β-ME, 2 mM TCEP, 1 mM PMSF, 1 × SIGMA*FAST* protease inhibitor cocktail; pH 7.5 at 4°C) and re-centrifuged at 150,000 × *g* for 90 min at 4°C. The pellets containing the microsomes were stored at −80°C.

### Detergent solubilisation of STRA6-GFP

Microsomes were resuspended in 50 mM HEPES, 300 mM NaCl, 10% glycerol, 10 mM β-ME, 1 mM PMSF and 1 × protease inhibitor cocktail; pH 7.5 [39]. The resuspended microsomes were then passed 3 times through a 23-gauge needle. An equal volume of solubilisation buffer (the above resuspension buffer containing twice the desired final concentration of detergent) was added to the resuspended microsomes and incubated overnight at 4°C with mixing. For the initial detergent screen, the final concentrations of detergents used for solubilisation were DDM (1%), LDAO (4.6%), DM (0.48%), FC-12 (0.7%), C12E9 (1%) and DDM: CHS (1%: 0.2%) [31]. For large-scale purification, 0.1 g of C12E9 per gram of original cell pellet was used to solubilize STRA6-GFP. Insoluble material was removed by centrifugation at 60,000 × *g* for 40 min at 4°C.

### Ni-NTA purification of STRA6-GFP

Solubilized membranes were supplemented with imidazole (final concentration 70 mM) and transferred to Ni-NTA (2.0 mL resin per 30 g of cells) that had been washed with dH_2_0 and pre-equilibrated for 40 min with Ni-NTA wash buffer (20 mM HEPES, 300 mM NaCl, 10% glycerol, 10 mM β-ME, 70 mM imidazole; pH 7.5) containing the appropriate detergent at the following final concentrations: DDM (0.05%), LDAO (0.28%), DM (0.19%), FC-12 (0.14%), C12E9 (0.05%) and DDM: CHS (0.05%: 0.01%). Solubilized membranes were incubated with Ni-NTA for 2 h at 4°C. The resin with bound STRA6-GFP was transferred to a column and washed with 30 CV of Ni-NTA wash buffer, 10 CV of high-salt Ni-NTA wash buffer (Ni-NTA wash buffer containing a further 300 mM NaCl) and a further 10 CV of Ni-NTA wash buffer. STRA6-GFP was eluted from the column with Ni-NTA elution buffer (as Ni-NTA wash buffer containing 250 mM imidazole, pH 7.5 at RT) in 0.5 CV fractions with 5 min between each elution.

### Fluorescence-detection size-exclusion chromatography (FSEC)

The “peak” fraction from the Ni-NTA eluate containing the majority of STRA6-GFP was identified using a blue light transilluminator (Clare Chemical Research) and centrifuged at 17,000 × *g* for 5 min at 4°C to remove particulate matter. The supernatant was removed and injected on either a Superose 6 or Superdex 200, 10/300 GL column (GE Healthcare) that had been pre-equilibrated with column buffer (as Ni-NTA wash buffer but without imidazole and reduced (4 mM) β-ME. The flow-rate of the column was maintained at 0.3 mL min^-1^ using an Äkta Purifier 100 system (Amersham Biosciences). Fractions were collected and subsequently analysed for fluorescence using a POLARstar Omega microplate reader (BMG labtech).

### Mass spectrometry

To confirm the identity of the isolated protein, peptide mass fingerprints were determined by mass spectroscopy. The in-gel digestion protocol of Shevchenko *et al*. (2006) was employed [47]. Briefly, the band was excised, cubed into 1 × 1 mm pieces and destained with 50 mM NH_4_HCO_3_ containing 50% acetonitrile for 30 min with occasional vortexing. An aliquot of 500 μL acetonitrile was added and incubated at room temperature with occasional vortexing until the gel pieces had become white and shrunk. The solution was then removed and replaced with 50 μL freshly-prepared trypsinization buffer (13 ng/μL trypsin in 10 mM NH_4_HCO_3_ containing 10% acetonitrile). Gel pieces were left on ice for 30 min and a further 10–20 μL aliquot of trypsinization buffer was added to cover the gel pieces where necessary. The gel pieces were left on ice for another 90 min to saturate them with trypsin and supplemented with 10–20 μL bicarbonate buffer (10 mM NH_4_HCO_3_ containing 10% acetonitrile) to keep them wet during enzymatic cleavage. Samples were then incubated at 37°C overnight. To extract the peptides from the gel pieces, aliquots of 180 μL extraction buffer (1:2 (v/v) 5% formic acid/acetonitrile) were added to each tube and incubated in a shaker at 600 RPM for 15 min at 37°C. The buffer was removed to a fresh Eppendorf and dried in a vacuum centrifuge. The peptides were then resuspended in 25 μL 0.1% formic acid by pipetting. For LC MS/MS analysis, the solution was filtered through a Spin-X 0.22 μm cellulose acetate centrifuge tube filter (Corning) at 12,000 × *g* for 5 min prior to injection.

Identification of peptides was performed with a Model 6340 Ion Trap LC/MS (Agilent Technologies, Dublin, Ireland). Proteins were identified from their peptide sequences, searching on the National Center for Biotechnology Information (NCBI; http://www.ncbi.nlm.nih.gov) non-redundant database. This database was interrogated using the MASCOT search engine (http://www.matrixscience.com; Matrix Science, London, UK). The search was performed against “All entries” in the taxonomic category, with carboxymethylated cysteines and oxidized methionines selected as fixed and variable modifications, respectively. Peptide tolerance was set to 1.2 Da and MS/MS tolerance was set to 0.6 Da.

### Expression and purification of His-RBP

The cloning and transformation of *Pichia pastoris* with His-RBP has been described previously [29]. After induction of RBP expression in the fermentor, the culture was centrifuged at 10,000 × *g* for 10 min and the supernatant collected. The supernatant was then incubated with Ni-NTA resin and His-RBP was purified as described previously [29]. The amount of His-RBP protein purified from a 6-L fermentor culture was approximately 1 g. Holo-RBP was prepared by incubation with an ethanolic solution of retinol for 10 min at 37°C to give a slight molar excess (approximately 1.1×) of retinol relative to RBP [29,44]. DyLight 594-conjugated RBP was prepared by mixing His-RBP with amine-reactive DyLight 594 dye according to manufacturer’s instructions. Unreacted fluor was removed from DyLight 594-conjugated RBP by size exclusion chromatography through a column of Sephadex G-25 (0.4 cm × 42 cm; medium grade; Sigma) that had been equilibrated with PBS.

### Confocal microscopy

Cells transformed with either empty vector or STRA6-GFP that had been induced for 48 h were harvested by centrifugation (700 × *g* for 5 min at 4°C) and resuspended in Krebs-Ringer buffer supplemented with 5 mM glucose. Equal amounts of cells (1 O.D. unit) were incubated with 85 μg DyLight 594-conjugated holo-RBP for 30 m at 4°C. The cells were then washed twice in 500 μL Krebs-Ringer buffer, resuspended in 200 μL, mounted on slides and analysed by confocal microscopy.

### Co-precipitation of RBP with STRA6-GFP

All steps were carried out at 4°C. Aliquots of broken cells derived from *Pichia pastoris* transformed with either empty vector or STRA6-GFP were incubated with 100 μg/mL holo-RBP for 10 min and centrifuged (12000 × *g* for 30 s). The supernatants were discarded and the pellets quickly resuspended in 50 μL lysis buffer (50 mM Tris-HCl, 150 mM KCl, 10 mM β-ME, 2 mM TCEP, 1 mM PMSF, 1 × SIGMA*FAST* protease inhibitor cocktail; pH 7.5 at 4°C) and re-centrifuged. After discarding the supernatants, the pellets were resuspended in 60 μL sample buffer, heated to 70°C for 10 min and centrifuged at 17,000 × *g* for 5 min. Samples were run on 10% SDS-PAGE gels and transferred to PVDF membranes, which were then immunoblotted using an anti-RBP antibody, an anti-c-Myc antibody (to detect STRA6-GFP) and an anti-actin antibody to confirm equal loading.

### Surface plasmon resonance

The surface of a sensor chip (CM5, GE Healthcare) was activated initially by mixing equal volumes (70 μL) of 400 mM ethyl(dimethylaminopropyl)-carbodiimide and 100 mM N-hydroxysulphosuccinimide and passing 80 μL of the mixture over the sensor surface at a flow rate of 10 μL/min. Rabbit anti-c-Myc polyclonal antibody (50 μg/mL in 10 mM NaOAc, pH 4.5) was immobilised onto the sensor chip at a flow rate of 10 μL/min for 20 min. Rabbit anti-HA polyclonal antibody (50 μg/mL in 10 mM NaOAc, pH 4.0) was immobilised onto the chip surface as a reference (negative control) using the same method applied to the anti-c-Myc polyclonal antibody. The surface was then capped with 1M ethanolamine HCl, pH 8.5, for 7 min, and further cleaned with 4 × 30 second injections of 10 mM NaOH (flow rate of 10 μL/min) removing any unbound or extraneous material. STRA6 (52.6 μg/mL, pH 7.4) was captured on the pre-immobilised surfaces with either anti-c-Myc or anti-HA antibodies at a flow rate of 5 μL/min for 2 min. Apo-RBP or holo-RBP (219.3, 109.7, 54.8, 27.4, 13.7 and 6.9 μM) was then passed over the STRA6 surface at a flow rate of 5 μL/ min and binding responses determined. The surface was regenerated using 15 μL of 10 mM NaOH. The 54.8 mM concentration of apo-RBP and holo-RBP was run in duplicate to check the stability of the assay and a zero concentration (using running buffer as blank) was included for double referencing. The assay was performed in triplicate and mean values determined. Association and dissociation phases were monitored for 4 and 10 min, respectively. All procedures were performed at room temperature. Equilibrium data were analyzed using GraphPad Prism 5 software.

## Supporting Information

S1 Fig Purification of RBP from *Pichia pastoris*. **(A)** Fed-batch fermentation of recombinant *Pichia pastoris* expressing RBP. Changes in the wet cell weight of yeast (red line) and dissolved oxygen (blue line) are shown over time. **(B)** Elution profile of Ni-NTA-purified RBP on a Superdex 200, 10/300 GL column. The void volume was determined using the elution profiles of blue dextran. **(C)** A PageBlue-stained 12% SDS gel showing 2 μg of Ni-NTA-purified RBP.
(TIF)

S2 Fig Solubilization of STRA6-GFP by selected detergents. Microsomes from *Pichia pastoris* expressing STRA6-GFP were resuspended and incubated overnight at 4°C in the following detergents: C12E9 (1%), DDM (1%), DDM:CHS (1%:0.2%), DM (0.48%), LDAO (4.6%) and FC-12 (0.7%). Insoluble material was removed by centrifugation (60,000 × *g* for 40 min at 4°C and the pellet resuspended to the original volume. The fluorescence of STRA6-GFP in the supernatants and resuspended pellets were measured and expressed as a percentage of the total fluorescence.
(TIF)
