# Supplementary figures and images for: Production of Functional Human Vitamin A Transporter/RBP Receptor (STRA6) for Structure Determination
Source: PLoS One. 2015 Mar 27;10(3):e0122293. doi: 10.1371/journal.pone.0122293 (PMC4376794; doi:10.1371/journal.pone.0122293)

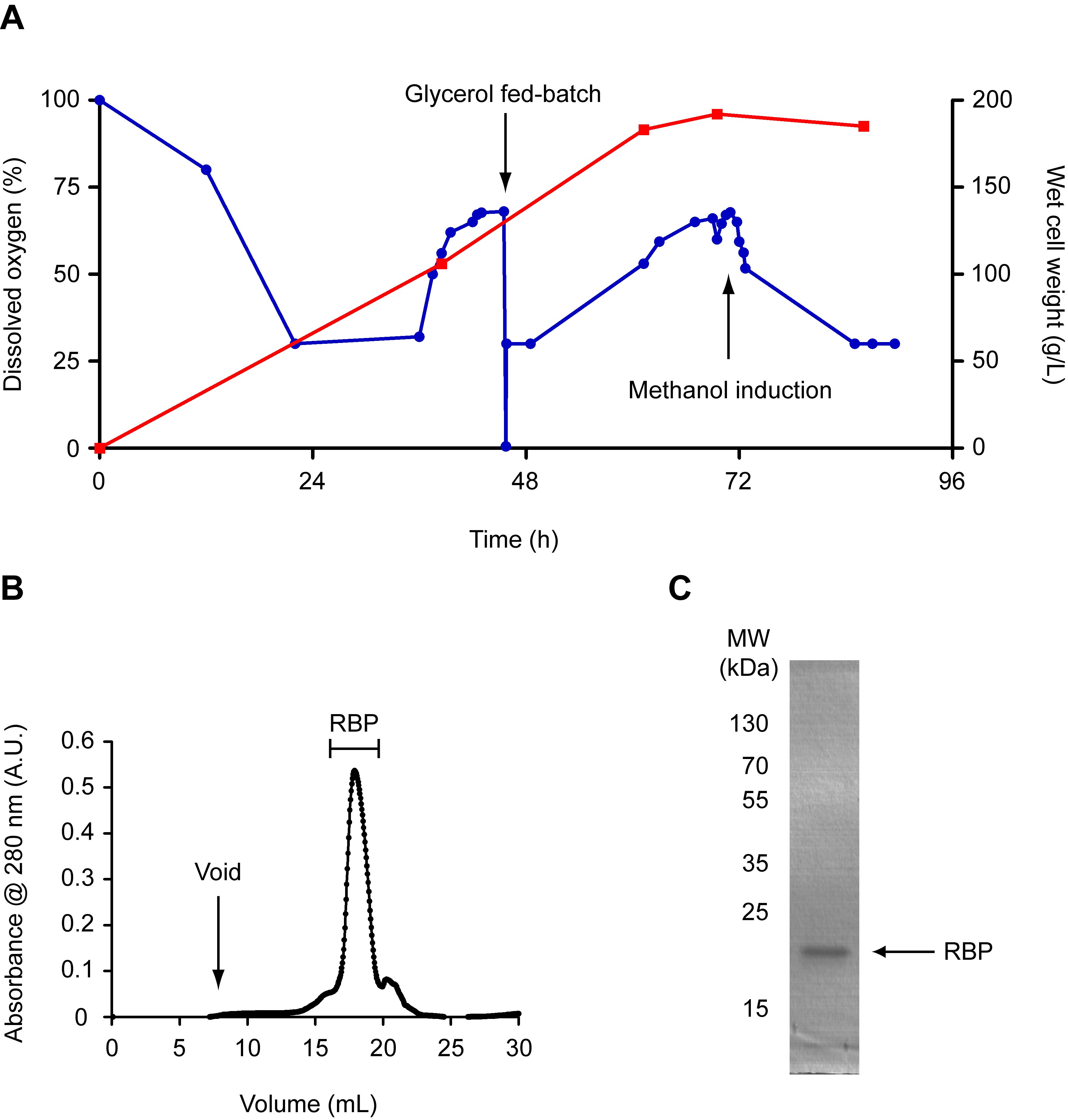

Supplement: S1 Fig — (A) Fed-batch fermentation of recombinant Pichia pastoris expressing RBP. Changes in the wet cell weight of yeast (red line) and dissolved oxygen (blue line) are shown over time. (B) Elution profile of Ni-NTA-purified RBP on a Superdex 200, 10/300 GL column. The void volume was determined using the elution profiles of blue dextran. (C) A PageBlue-stained 12% SDS gel showing 2 μg of Ni-NTA-purified RBP. (TIF) [file pone.0122293.s001.tif]

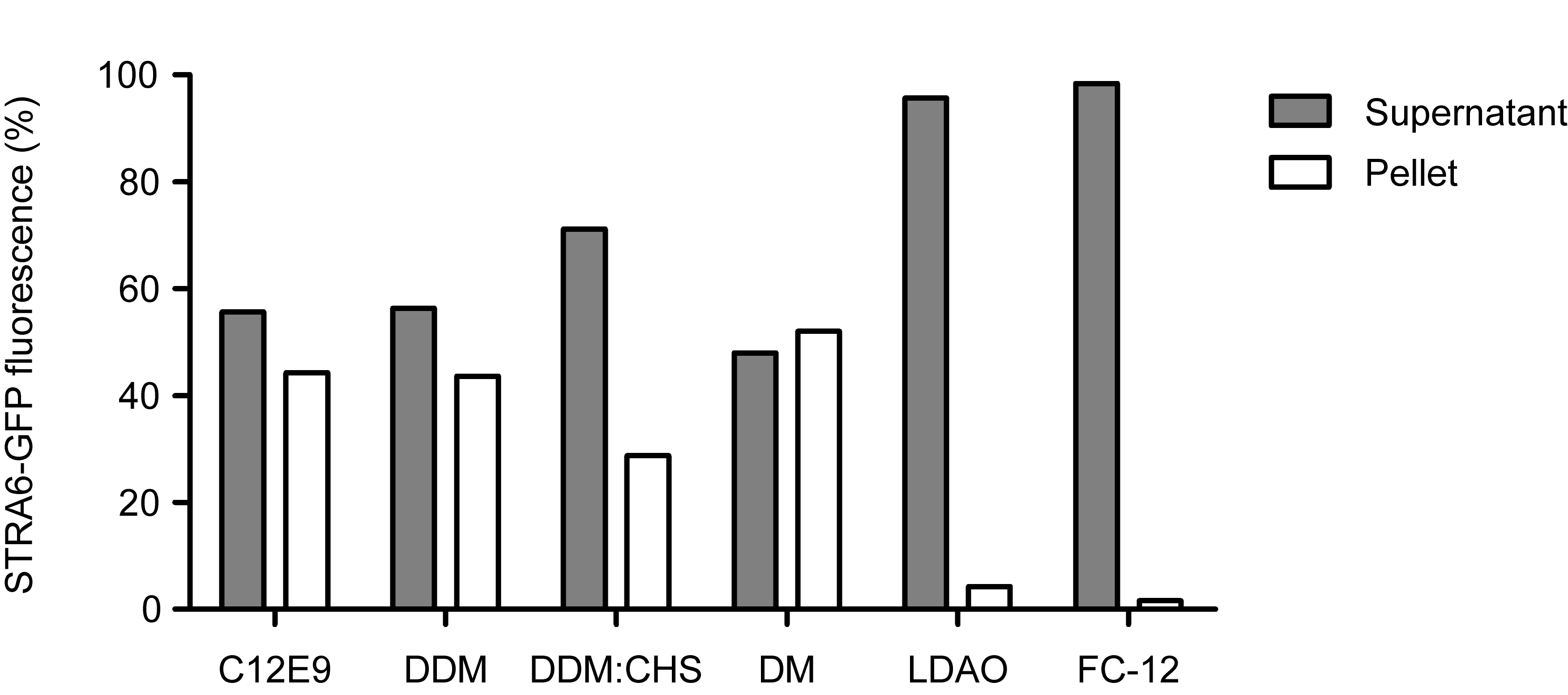

Supplement: S2 Fig — Microsomes from Pichia pastoris expressing STRA6-GFP were resuspended and incubated overnight at 4°C in the following detergents: C12E9 (1%), DDM (1%), DDM:CHS (1%:0.2%), DM (0.48%), LDAO (4.6%) and FC-12 (0.7%). Insoluble material was removed by centrifugation (60,000 × g for 40 min at 4°C and the pellet resuspended to the original volume. The fluorescence of STRA6-GFP in the supernatants and resuspended pellets were measured and expressed as a percentage of the total fluorescence. (TIF) [file pone.0122293.s002.tif]
